# Zinc in Inflammatory Bowel Disease: Biological Functions, Clinical Associations and Therapeutic Uncertainty

**DOI:** 10.3390/nu18213575

**Published:** 2026-10-29

**Authors:** Richard Hall, Michael Colwill, Aaron S. Bancil, Kamal V. Patel, Sailish Honap, Andrew Poullis

**Affiliations:** 1 Department of Gastroenterology, St George’s University Hospitals NHS Foundation Trust, London SW17 0QT, UKandrew.poullis@stgeorges.nhs.uk (A.P.); 2 Institute of Infection and Immunity, City St George’s, University of London, London SW17 0RE, UK; 3 School of Immunology and Microbial Sciences, King’s College London, Strand, London WC2R 2LS, UK

**Keywords:** inflammatory bowel disease, ulcerative colitis, Crohn’s disease, zinc, zinc deficiency, micronutrients

## Abstract

Zinc deficiency is reported in up to half of patients with inflammatory bowel disease (IBD), yet interpreting a low serum zinc concentration can be challenging. Zinc is an essential micronutrient with important roles in epithelial barrier integrity, immune regulation and cellular signalling, providing biological plausibility that deficiency could contribute to intestinal inflammation. Observational studies have associated low serum zinc with adverse outcomes, including hospitalisation, surgery and relapse, particularly in Crohn’s disease. However, both systemic inflammation and hypoalbuminaemia reduce circulating zinc concentrations, raising uncertainty as to whether low zinc represents a true deficiency, a marker of active disease or a potentially modifiable contributor to inflammation. This narrative review examines the mechanistic, observational and interventional evidence linking zinc with IBD. Experimental studies support effects on tight-junction integrity, mucus production, Paneth cell function and immune signalling. Despite this biological rationale and the associations seen in observational studies, evidence that zinc supplementation improves IBD-related outcomes remains limited. Existing studies are small, heterogeneous and often rely on surrogate outcomes, while oral supplementation has not consistently improved clinical or inflammatory endpoints. Zinc deficiency should be identified and corrected as part of nutritional care in IBD but serum zinc concentrations should be interpreted in the context of underlying inflammation, and the benefit of routine zinc monitoring remains uncertain. Well-designed prospective trials are needed to determine whether correcting zinc deficiency improves objective IBD outcomes.

## 1. Introduction

Inflammatory bowel disease (IBD), comprising ulcerative colitis (UC) [1] and Crohn’s disease (CD) [2], is a chronic immune-mediated disorder characterised by intestinal inflammation. IBD typically presents in early adulthood and is caused by an interplay between genetic susceptibility, immune dysregulation, dysbiosis and environmental exposures [3]. Although advances in biologic and small molecule therapies have transformed treatment, many patients continue to experience symptoms, complications, hospitalisation and surgery.

Nutritional deficiencies are amongst the most common complications of IBD and may occur in both active and quiescent disease [4]. The causes of nutritional deficiencies in IBD are multifactorial and include reduced dietary intake, malabsorption, intestinal losses, systemic inflammation, surgery and/or increased metabolic demands [5]. Deficiencies in micronutrients such as iron, vitamin D, folate and vitamin B12 are well recognised and often prioritised in routine practice [6,7]. Trace elements, such as zinc, are less consistently assessed, despite evidence that zinc deficiency may affect half of patients with IBD [8] and guidelines recommending patients should be monitored regularly for micronutrient deficiencies [9].

Zinc is an essential trace element that has many important roles in the human body, including growth, immune function, cellular signalling, gene transcription and tissue repair. Within the gastrointestinal tract, preclinical models have demonstrated that zinc deficiency disrupts tight junctions, which can impair intestinal permeability, impair Paneth cell function, promote dysbiosis and amplify proinflammatory cytokines [10]. These pathways overlap with mechanisms implicated in IBD pathogenesis, making zinc plausibly not only a marker of nutritional deficiency, but also a potential contributor to intestinal inflammation [10].

Despite the mechanistic plausibility and high prevalence of zinc deficiency seen in IBD, the clinical evidence is mixed. Observational studies have associated zinc deficiency with adverse clinical outcomes, including disease activity, hospitalisation and surgery [11,12], but these findings are not universal [13], and such study designs are unable to establish causality. Interpretation of serum zinc is also imperfect as it represents a small proportion of total body zinc and is heavily influenced by systemic inflammation and hypoalbuminaemia, both factors that are highly relevant in IBD.

The central uncertainty is whether low zinc in IBD represents an underlying deficiency capable of contributing to intestinal inflammation, a consequence of active disease and malnutrition, or a surrogate marker of inflammation. Establishing this distinction is important in determining the clinical significance of zinc status in IBD.

Recent reviews have explored the role of zinc in IBD, focusing on zinc homeostasis, transporter biology and molecular mechanisms [14]. The present narrative review will adopt a more clinically focused perspective, examining the biological rationale, clinical association and evidence for zinc supplementation in IBD. A particular emphasis will be placed on the association between zinc status and disease outcomes, the interpretation of zinc status and the evidence informing zinc supplementation in clinical practice.

## 2. Methods

A literature search was conducted on PubMed/MEDLINE from database inception to 14th May 2026. Search terms included, alone or in combination, “zinc”, “zinc deficiency”, “hypozincemia”, “zinc acetate”, “zinc sulphate”, “zinc gluconate”, “zinc transporter”, “ZIP4”, “ZnT1”, “inflammatory bowel disease”, “Crohn’s disease”, “ulcerative colitis”, “tight junction”, “permeability”, “mucosal healing”, “colitis”. Reference lists of key papers were manually screened for additional relevant publications.

Original research studies, systematic reviews, meta-analyses, narrative reviews and clinical guidelines published in English were considered. Studies were selected according to their relevance to the objective of this narrative review, with priority given to human studies examining zinc status, disease activity, clinical outcomes and/or zinc supplementation in IBD. Preclinical studies were included where they provided insight into the mechanisms through which zinc may impact intestinal barrier function, immune regulation and inflammation. The evidence was reviewed and synthesised narratively, considering study design, sample size, patient characteristics, reported outcomes and potential sources of confounding. Mechanistic studies were used to establish biological plausibility. Observational studies were evaluated in the context of their clinical relevance, with particular attention given to the limitation of inferring causality from associations. For supplementation studies, consideration was given to differences in study populations, zinc formulation, treatment duration and relevance of study endpoints. Given the narrative nature of the review, no formal risk-of-bias assessment or quantitative meta-analysis was undertaken. Study selection and inclusion were based on author judgement rather than a systematic screening process, introducing the possibility of selection bias and incomplete identification of relevant evidence.

## 3. Zinc

### 3.1. Zinc Intake, Absorption and Homeostasis

Zinc is an essential trace element that humans cannot endogenously produce or store in substantial amounts and therefore must be regularly obtained from diet. Dietary sources include meat, fish, seafood, eggs, dairy, fortified breakfast cereals as well as plant-based foods such as beans, nuts, seeds and grains, particularly chickpeas, lentils, pumpkin seeds and sesame seeds. Diet also has an important effect on zinc bioavailability. Phytate, found in cereals, legumes and seeds, can impair zinc absorption as it binds with zinc, forming poorly soluble complexes within the intestinal lumen, subsequently reducing bioavailability [15]. Polyphenols, present in plant-based foods and beverages such as tea and coffee, may also impair zinc absorption through metal complex formation, although human evidence remains limited, and the effect appears dependent on the associated dietary matrix [16,17]. On the other hand, dietary protein from sources such as meat can improve zinc absorption by increasing luminal zinc solubility through amino acid and peptide binding. Food preparation methods such as fermentation, soaking or germination can reduce total phytate content and improve bioavailability [15].

The recommended daily intake of zinc varies by country, with the US recommending 11 mg per day in adult men and 8 mg per day for women, and the UK recommending 9.5 mg/day for men and 7.0 mg/day for women [18]. The human body contains between 2 and 3 g of zinc that is mainly stored in skeletal muscle (55–60%), bone (30%) and smaller quantities in the skin, liver, pancreas, kidney, retina and prostate. Serum or plasma zinc accounts for less than 1% of total body zinc, which is clinically important when assessing zinc status [19].

**Figure 1:**
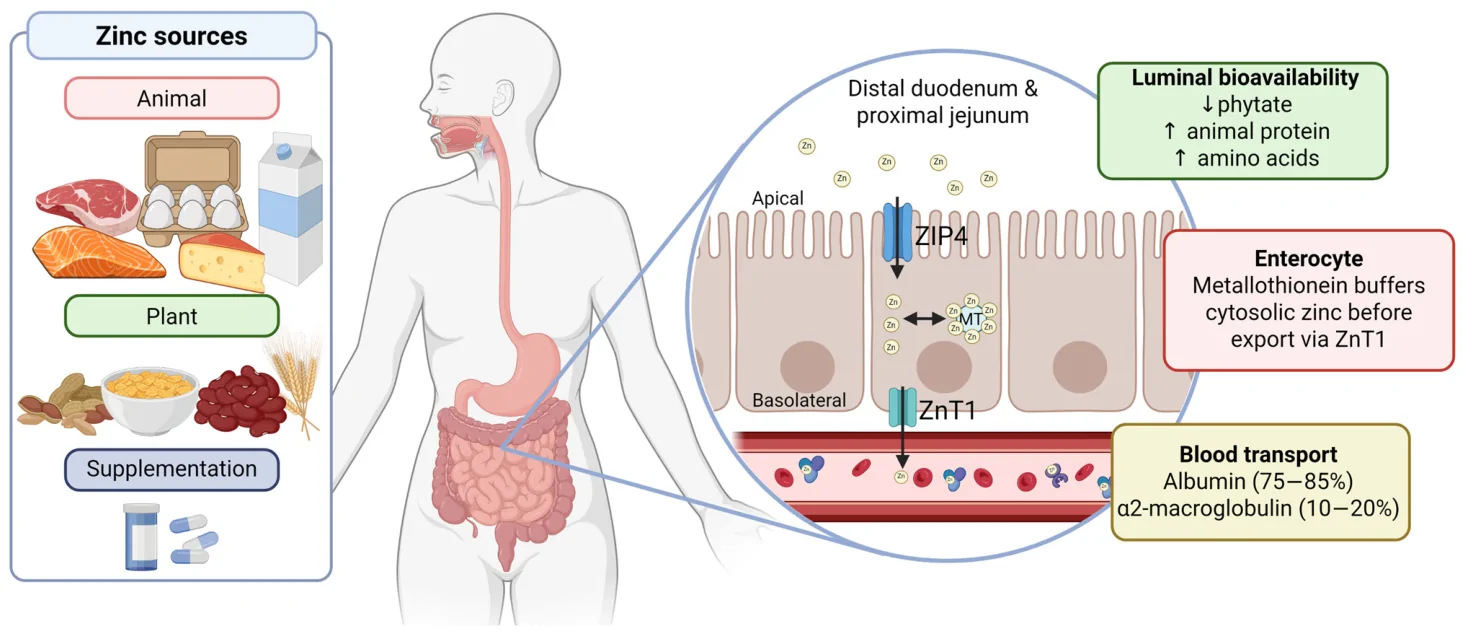
Dietary sources, intestinal absorption and transport of zinc. Zinc is obtained from dietary sources and supplementation and is absorbed predominantly in the distal duodenum and proximal jejunum. ZIP4 mediates apical uptake, ZnT1 basolateral export, and metallothionein buffers intracellular zinc. Circulating zinc is transported mainly bound to albumin and α2-macroglobulin. MT, metallothionein; ZIP4, Zrt-/Irt-like protein 4; ZnT1, zinc transporter 1. Created with BioRender.com. Hall, R. (2026).

Following oral ingestion, zinc is released as ionised zinc or zinc complexed with ligands such as amino acids, organic acids or phytate. As outlined in Figure 1, zinc is predominantly absorbed in the small intestine, particularly the distal duodenum and proximal jejunum. Absorption across the enterocyte is tightly regulated by two main families of transporter proteins, Zinc-regulated transporter and Iron-regulated transporter (Zrt-/Irt)-like proteins (ZIPs), which increase cytosolic zinc concentration by transporting zinc into the cell, and Zinc Transporter (ZnT) proteins that reduce cytosolic zinc by transporting zinc out of the cell or into intracellular compartments [14]. In enterocytes, the predominant apical transporter is ZIP4, which transports zinc from the lumen into the cell, whilst ZnT1 is important for basolateral export into the portal circulation [19]. Following zinc uptake into the enterocyte, it can be used locally, bound intracellularly or exported across the basolateral membrane into the portal circulation. These processes are tightly regulated, and when zinc concentrations are low, expression of ZIP4 can be upregulated, leading to increased absorption. Metallothioneins are central to this regulation as they bind zinc with a high affinity and act as an intracellular buffer [20]. By sequestering intracellular zinc, metallothioneins limit zinc toxicity and regulate the amount of zinc that is ultimately released into the circulation. Once zinc enters the portal circulation it is transported in plasma largely bound to albumin and α2-macroglobulin [19]. Zinc is excreted predominantly through the gastrointestinal tract, comprising unabsorbed dietary zinc, zinc within shed enterocytes and endogenously secreted zinc from pancreatic and intestinal secretions [21].

### 3.2. Biological Functions of Zinc

Zinc has a broad range of roles in human physiology, as it functions as a structural, catalytic and regulatory ion. It is estimated that zinc binds to approximately 10% of all proteins in the human proteome, including many enzymes and transcription factors [22]. As a structural ion, zinc stabilises proteins, and zinc finger proteins mediate interactions with DNA, RNA and other proteins. Through these mechanisms zinc has a role in gene transcription, DNA repair, protein synthesis and cell division [23,24].

Zinc also acts as an intracellular signalling ion. Cellular zinc is tightly regulated, allowing transient changes in zinc concentrations to alter cell signals involved in immune activation, cytokine production, oxidative stress and cell survival [25,26]. Additionally, zinc is essential for tissue repair and wound healing, contributing to haemostasis, inflammation, reepithelialisation, angiogenesis, collagen synthesis and extracellular matrix remodelling [27]. Zinc-dependent enzymes, including matrix metalloproteinases, participate in these processes, whilst zinc also promotes epithelial cell proliferation and migration.

Given the broad cellular functions of zinc, zinc deficiency has been associated with a wide range of human pathology [28]. Zinc has a role in bone metabolism, pancreatic β-cell function, insulin storage, thyroid hormone metabolism, male fertility, central nervous system development and retinal immunology [29]. Zinc has also been demonstrated to play a role in mediating intestinal inflammation and maintaining intestinal barrier integrity, which is likely to be central to its potential role in IBD [10].

### 3.3. Zinc Deficiency

Zinc deficiency is common globally, affecting up to 17% of the world’s population [30], and may occur through several mechanisms [31]. Inadequate dietary intake is the most common cause globally, particularly in low- and middle-income countries where diets are reliant on cereals and legumes, which are high in phytate [30,32]. Dietary deficiency alone is less common in developed countries; however, additional risk factors such as pregnancy, breastfeeding and restrictive diets often coexist. Additional causes of zinc deficiency include malabsorption, chronic diarrhoea, intestinal failure, alcohol excess, bariatric surgery, parenteral nutrition and diabetes. Acrodermatitis enteropathica represents an autosomal recessive inherited condition caused by a pathogenic variant SLC39A4 encoding ZIP4, which leads to severe zinc deficiency due to impaired intestinal absorption [31].

Clinically, mild zinc deficiency is often asymptomatic or present with non-specific symptoms. Severe deficiency manifests as dermatitis, alopecia, diarrhoea, anorexia, impaired wound healing, taste disturbance, infections and, in children, impaired growth and development [33].

Zinc supplementation is available in several formulations, including zinc oxide, sulphate, gluconate, acetate and citrate. These preparations differ in elemental zinc content and absorption profiles, which influences zinc bioavailability. A recent review suggested that zinc glycinate and zinc gluconate are well absorbed, although the authors are cautious in their conclusions given the small number of studies and heterogeneity [34]. Zinc absorption may also be influenced by administration with food, particularly phytate-rich meals, with some studies demonstrating greater absorption when supplements are taken in a fasted state [35,36,37]. However, fasting administration may be less well tolerated, and both the optimal timing of supplementation and the clinical significance of these differences remain uncertain. Short-term supplementation is generally well tolerated, although gastrointestinal adverse effects including nausea, abdominal discomfort, vomiting and diarrhoea may occur. Prolonged or high-dose zinc supplementation can impair copper absorption, leading to copper deficiency, which usually results in anaemia, neutropenia and, less commonly, neurological manifestations [38].

### 3.4. Blood Zinc Levels

**Figure 2:**
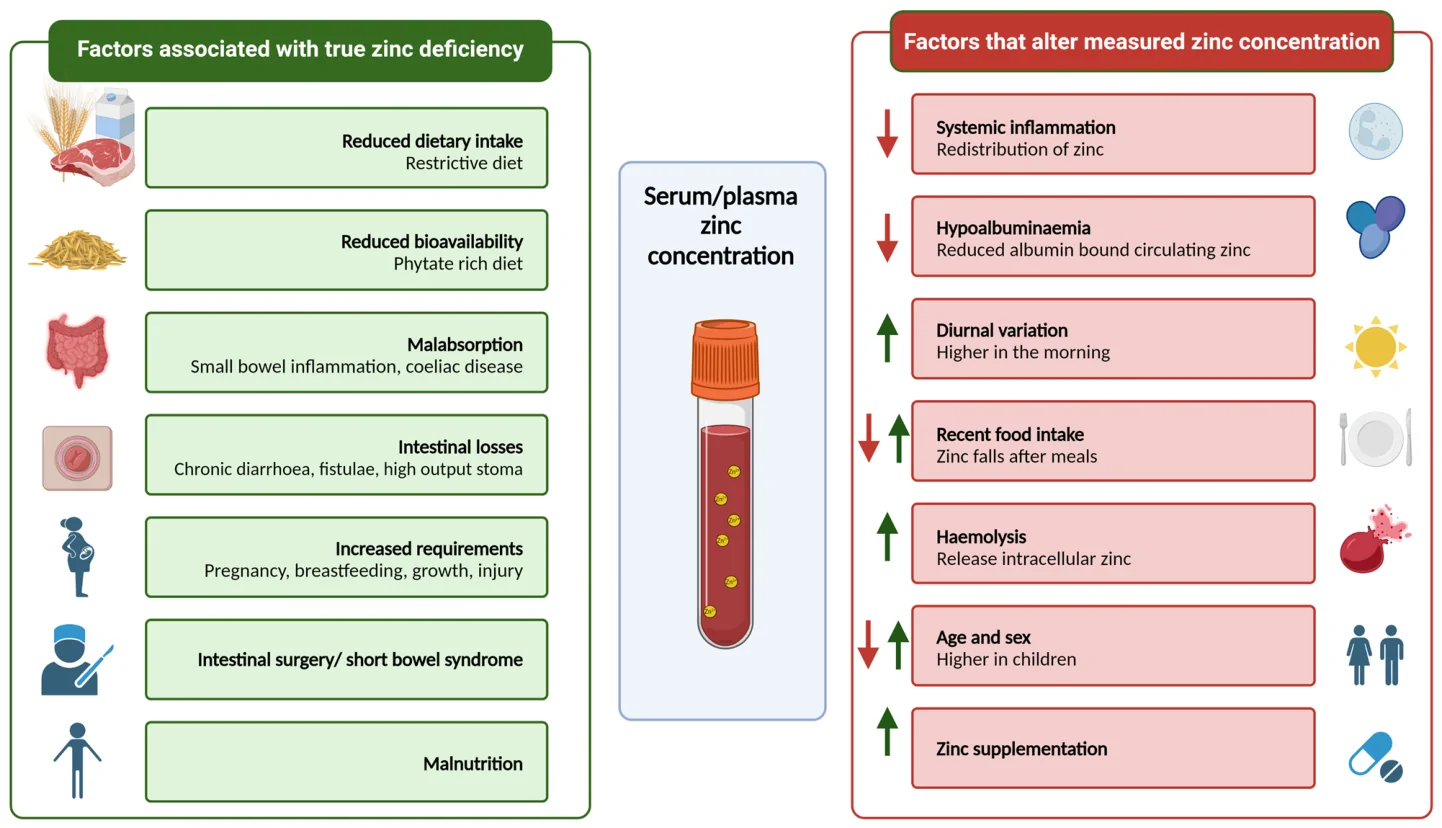
Factors influencing serum and plasma zinc concentrations. Clinical interpretation of circulating zinc can be challenging due to several biological and clinical factors. Created with BioRender.com. Hall, R. (2026).

When interpreting zinc studies, it is important to recognise the limitations that exist in current methods. In clinical practice, zinc status is usually assessed using serum or plasma zinc concentrations; these are often used interchangeably, despite subtle differences [39]. However, neither are markers of total zinc stores, and they represent < 1% of total body zinc [19]. As outlined in Figure 2, zinc concentrations are also influenced by factors including fasting status, diurnal variation, recent intake, age, sex and supplementation [39]. Haemolysis may also affect measurement, as zinc is present in much higher concentrations intracellularly.

Importantly in IBD, inflammation and hypoalbuminaemia are important confounders [40]. During the acute phase response plasma and serum zinc concentrations fall, probably reflecting redistribution of zinc from plasma into other tissues [41]. Additionally, a majority of the circulating zinc is bound to albumin, meaning that in hypoalbuminaemic states zinc concentrations may fall independently of whole body zinc stores [39]. As inflammation and hypoalbuminaemia are both common in active IBD, a low serum or plasma zinc concentration may reflect true deficiency, inflammatory redistribution or hypoalbuminaemia, or a combination of these. Interpreting low serum or plasma zinc in IBD therefore requires caution and should be considered alongside CRP, albumin, markers of disease activity, diarrhoea, surgical history and supplementation history. Approaches to adjust circulating zinc concentrations for inflammation have been proposed, including the Biomarkers Reflecting Inflammation and Nutritional Determinants of Anaemia (BRINDA) project. BRINDA proposed a regression-based adjustment of serum or plasma zinc concentrations according to inflammatory biomarkers such as CRP. However, this framework was developed primarily for population-level nutritional assessment and has not been validated for use in patients with IBD [42].

Alternative measures of zinc status such as hair and urine concentrations have been explored but are not routinely used in clinical practice [43]. There are currently no widely available alternative non-invasive tests, and therefore serum or plasma zinc remains the most practical clinical biomarker. Despite its limitations, circulating zinc concentrations have been shown to respond to changes in dietary intake and supplementation, and zinc assessment remains supported by international guidance as part of nutritional care in IBD [9,43,44]. However, a low circulating zinc concentration does not necessarily indicate a clinically significant deficiency or, in isolation, justify supplementation. The appropriate management of isolated low circulating zinc concentrations, particularly in patients with active inflammation, remains uncertain. Furthermore, there is insufficient evidence that routine zinc monitoring improves clinical outcomes in IBD, or that correction of low circulating zinc concentrations alters disease progression.

### 3.5. Zinc, Intestinal Barrier Dysfunction and Immune Activation

Zinc has several biological functions that are directly relevant to intestinal inflammation. It contributes to the intestinal barrier function through effects on epithelial integrity, tight junctions, and mucin production, and also has a role in immune signalling [10]. Given these pathways are also implicated in IBD, they provide a plausible mechanism through which zinc deficiency could contribute to intestinal inflammation, although much of the evidence is preclinical.

#### 3.5.1. Intestinal Barrier

The intestinal mucosal barrier forms the interface between the luminal contents, commensal bacteria and host immune system. It consists of a mucus layer, anti-microbial peptides, immunoglobulin, a single layer of intestinal epithelial cells linked by tight-junction complexes and an innate and adaptive immune system [45]. Its role is to facilitate the absorption of nutrients and water, whilst at the same time preventing the passage of toxins and defending against pathogens. Barrier dysfunction is well described in the pathogenesis of IBD [45,46].

Experimental studies have suggested that zinc is important for epithelial tight-junction integrity. In Caco-2 monolayers, depletion of intracellular zinc using a zinc chelator reduced expression of tight-junction proteins claudin-3 and occludin. This was associated with impaired barrier function, demonstrated by increased Fluorescein Isothiocyanate-Dextran(FITC)-dextran flux [47]. Other Caco-2 models have similarly shown that zinc deficiency can disrupt tight-junction proteins and is associated with subsequent increased neutrophil transmigration [48]. Conversely, zinc supplementation can modify tight-junction composition and improve some measures of epithelial barrier function, although effects on paracellular permeability appear selective [49]. Further in vitro studies suggest that zinc supplementation may enhance barrier integrity through PI3K/AKT/mTOR signalling, with increased transepithelial electrical resistance, reduced tight-junction permeability and increased Zonula Occludens (ZO)-1 expression [50].

Zinc transporter models provide additional evidence that zinc is required for intestinal epithelial integrity. In ZIP4 knockout mice, loss of the apical zinc transporter resulted in rapid intestinal zinc depletion and disruption of intestinal integrity, unless mice were nursed or given excess dietary zinc [51].

Zinc has also been found to alter the mucus layer. In HT-29-MTX human colorectal adenocarcinoma-derived goblet cell models, zinc deficiency impaired mucin production and secretion, with an effect on both MUC2 and MUC5AC mRNA expression. These effects led to disturbed post-translational mucin O-glycosylation, altering mucin stability [52].

Zinc may also contribute to antimicrobial barrier function through effects on Paneth cells. Podany et al. [53] demonstrated that ZnT2-mediated zinc transport into Paneth cell granules is required for normal secretory granule function. ZnT2 knockout mice had hypodense secretory granules, reduced active lysozyme, autophagosome accumulation and granule degradation, suggesting impaired antimicrobial secretion. This is particularly relevant in CD, where Paneth cell dysfunction has been implicated in ileal disease [54].

Animal colitis models provide further support for a barrier protective role for zinc. In an acetic acid-induced rat model of colitis, zinc and zinc picolinate reduced colonic injury, oxidative stress and inflammatory markers whilst improving glutathione and tight-junction protein expression [55]. In a Trinitrobenzene Sulfonic (TNBS)-induced mouse model, dietary zinc oxide nanoparticles reduced mucosal injury, inflammation and crypt damage, with reported effects on goblet cell function, tight-junction proteins, epithelial proliferation and gut microbiota diversity [56].

Direct human data is very limited. In a small uncontrolled study of 12 patients with quiescent CD and increased intestinal permeability, oral zinc supplementation improved lactulose/mannitol ratios, a measure of intestinal permeability [57]. However, these findings have not been independently replicated.

#### 3.5.2. Immune-Inflammatory Pathways

Zinc deficiency may also amplify intestinal inflammation independently of epithelial barrier impairment, as it has a role in innate and adaptive immune functions [26].

The interleukin (IL)-23/Th17 pathway provides strong mechanistic overlap between zinc deficiency and IBD. IL-23 promotes Th17 responses, which is central to chronic intestinal inflammation [58]. In a mouse model, zinc deficiency exacerbated experimental colitis, promoting upregulation of IL-23p19 expression in macrophages in the lamina propria, and promoting activation of the IL-23/Th17 axis [59]. Supporting in vitro work from the same study also demonstrated increased Il-23p19 expression in bone marrow-derived macrophages, supporting a direct effect of low zinc on macrophage inflammatory signalling [59].

Animal studies have also demonstrated that zinc deficiency can increase tumour necrosis factor (TNF)-α mediated inflammation [60,61], another key mediator of intestinal inflammation [62]. In rats with DSS-induced colitis, Iwaya et al. [61] demonstrated that moderate dietary zinc deficiency worsened colitis, caused neutrophils to accumulate in the colonic mucosa and increased TNF-α production, despite no significant differences observed in colonic permeability. Similar findings were reported by Suwendi et al. [60], who reported that zinc deficiency worsened dextran sodium sulphate (DSS)-colitis and increased TNF-α production. These studies support the hypothesis that zinc can exacerbate inflammation directly through immune-mediated pathways rather than via intestinal permeability.

Metallothionein models provide further support. In DSS-induced colitis, metallothionein-I/II knock out mice developed more severe colonic injury, higher inflammatory-cell infiltration and higher mucosal TNF-α, interferon (IFN)-γ and IL-17. Macrophages from these mice also produced more pro-inflammatory cytokines, suggesting that impaired intracellular zinc buffering may amplify macrophage-mediated inflammation [63].

Supplementation studies have complemented the findings that zinc can modulate immune responses in experimental colitis. In mice with TNBS-induced colitis, dietary zinc improved colitis symptoms, increased CD3+, CD3+CD8+, and Th2 cells, suppressed Th1 and Th17 cells, and decreased the production of serum IL-1β and IL-18 [64].

Overall, experimental evidence supports a role for zinc in maintaining intestinal barrier integrity and regulating mucosal immune responses. These mechanisms provide plausibility that zinc deficiency may contribute to IBD, although the mechanistic evidence remains predominantly preclinical, and causality in humans remains unproven.

## 4. Zinc and Inflammatory Bowel Disease

### 4.1. Dietary Zinc Intake and Incident IBD

Given that zinc has several plausible mechanisms that are relevant to IBD pathogenesis, it has been proposed that zinc deficiency may influence susceptibility to developing IBD.

In the prospective Nurses’ Health Study I & II cohorts, 170,776 women were followed up for 26 years to examine the association between zinc intake and incident IBD [65]. Zinc intake was assessed using food frequency questionnaires and analysed in relation to the subsequent development of IBD. Higher zinc intake was associated with a lower risk of developing CD, particularly for higher dietary intake rather than supplemental zinc. In analysis of a cumulative long-term zinc intake the multi-variate hazard ratio of developing CD was 0.64 [95% CI, 0.42–0.97] and 0.71 [95% CI, 0.46–1.1] in the fourth and fifth quintiles compared to the lowest quintile. The association was not dose dependent, with the benefit appearing to plateau at moderate intake. There were no clear protective associations seen with supplemental zinc, and zinc intake was not associated with a risk of developing UC. The study is limited with the inclusion of women only, predominantly Caucasian ethnicity and reliance on food-frequency questionnaires rather than measured zinc status.

Similarly, in the prospective Nutri-Sante cohort [66], dietary zinc intake was assessed using 24 h dietary records at 6-month intervals and analysed in relation to incident IBD cases. Among the 105,832 participants, 27 incident CD and 48 incident UC cases were identified. They observed that dietary zinc intake was inversely associated with risk of incident CD, in adjusted Poisson models, with the relative risk 0.60 [95% CI, 0.22–1.66] in tertile 1 and 0.12 [95% CI, 0.02–0.73] in tertile 2. There was no significant association between zinc intake and the risk of developing UC.

In contrast to the signal for CD, evidence in UC appears to be less clear. A meta-analysis of observational studies investigating micronutrient intake and UC found no association between zinc intake and the risk of developing UC [67]. In contrast, a Japanese hospital-based case-control study [68] found higher dietary zinc intake one year before diagnosis was associated with lower odds of developing UC [OR 0.39, 95% CI, 0.18–0.85]. However, this study is limited by its retrospective dietary assessment, small sample size, potential for recall bias and case-control design.

Overall, current evidence appears to support the hypothesis that higher dietary zinc intake may be associated with a lower risk of developing CD. However, the available data are observational and dependent on dietary recall, which limits the conclusions that can be drawn. Reported dietary zinc intake could reasonably represent higher quality of diet overall rather than zinc intake alone. No study is yet to establish zinc deficiency as a causal risk in the development of IBD.

### 4.2. Prevalence of Zinc Deficiency in IBD

Serum zinc deficiency is common in patients with IBD, although reported prevalence varies substantially between studies. This variability reflects differences in populations, age, disease phenotype, disease activity, nutritional status, assays used and the thresholds used to define deficiency.

A systematic review and meta-analysis of nine published articles comprising 2413 patients with known IBD estimated that the pooled prevalence of zinc deficiency in IBD was 50% [95% CI, 0.48–0.52] [8]. Prevalence of zinc deficiency was higher in CD [54%, 95% CI, 0.51–0.56] than UC [41%, 95% CI 0.38–0.45]; however, heterogeneity was substantial, with an *I*^2^ of 96%, and individual study estimates ranged from 11.2% to 88%. In a more recent meta-analysis including seven adult-only studies [69], pooled zinc deficiency prevalence was reported at 35% [95% CI, 0.19–0.52], again with substantial heterogeneity (*I*^2^ = 98.5%). Serum zinc deficiency was more common in CD than UC, with pooled estimates of 40% [95% CI, 0.21–0.59] and 33% [95% CI, 0.18–0.51] respectively.

Individual cohort studies have demonstrated similar variation. In a retrospective study of 447 patients zinc deficiency was 45.6% overall, including 48.3% in CD and 36% in UC [70]. This cohort had a high burden of aggressive disease, which may partly explain the high prevalence. However, zinc deficiency was also reported in 30% of patients in clinical remission and 45% of patients in endoscopic remission, suggesting that low serum zinc may persist when inflammatory activity appears to be controlled. Siva et al. [11] reported zinc deficiency in 42.1% in CD and 38.5% in UC in a large registry cohort. By contrast, Brownson et al. [71] reported a lower prevalence of 12.9% amongst patients receiving biologic therapy in data from a single centre.

Overall, serum zinc deficiency appears common in IBD patients, although estimates vary substantially according to the populations studied. Reported prevalence appears higher in CD than UC. However, it remains unclear if the measured zinc deficiency represents a true deficiency or a surrogate marker of inflammation or hypoalbuminaemia.

### 4.3. Zinc Deficiency and Adverse IBD Outcomes

Several observational studies have suggested that zinc deficiency is associated with more adverse outcomes in IBD, although the strength and consistency of this association varies, and the association appears stronger in CD than UC. In one of the largest studies to date, Siva et al. [11] analysed 773 patients with CD and 223 patients with UC from a prospectively maintained registry. In CD, zinc deficiency was associated with an increased risk of hospitalisation [OR 1.44, 95% CI 1.02–2.04, *p* = 0.04], surgery [OR 2.03, 95% CI 1.36–3.02 *p* = 0.001] and disease-related complications [OR 1.50, 95% CI 1.04–2.15, *p* = 0.03], whereas in UC, zinc deficiency was associated with an increased risk of hospitalisation [OR 2.14, 95% CI 1.07–4.29]; however, both IBD-related surgery and complications were not significantly associated. Normalisation of zinc within 12 months was associated with improved outcomes in both CD and UC, suggesting that either zinc is a modifiable risk factor in IBD or that improving zinc levels are a reflection of improvement in underlying inflammation. Conclusions from this observational study should be made cautiously.

In a more recent retrospective cohort of 447 patients with IBD, zinc deficiency was identified in 45.6% of patients. Zinc deficiency was associated with higher CRP and faecal calprotectin, along with higher corticosteroid use, IBD-related hospitalisation, IBD-related surgery and IBD-related complications [70]. These associations persisted after adjustment for inflammatory markers, although subgroup analysis suggested the signal was driven by patients with CD rather than UC.

Associations between serum zinc and contemporaneous disease activity have been inconclusive. Omatsu et al. [72] reported that in CD, serum zinc deficiency was associated with clinical, biochemical and endoscopic disease activity, whereas in UC it was associated with endoscopic activity, though not CRP or the Lichtiger index. Zinc also correlated with albumin and Onodera’s prognostic nutritional index, suggesting that zinc deficiency reflects both inflammatory burden and nutritional status. In contrast, Mechie et al. [73] found zinc deficiency was uncommon when using a lower threshold of 7 µmol/L (6% of 232 patients) and was not associated with either clinical or deep remission in CD or UC. Both studies are limited by their small sample sizes, single centre design and low prevalence, limiting statistical power. These findings also highlight the importance of using an appropriate zinc threshold to define deficiency.

Data in clinically quiescent IBD is also mixed. In a prospective study of 93 patients, MacMaster et al. [12] found that zinc deficiency in quiescent IBD was associated with a shorter time to relapse [HR 6.9, 95% CI 1.9–26, *p* = 0.008], with a stronger association in CD than UC. In contrast a Swiss retrospective cohort study following 97 patients in clinical remission did not find that baseline zinc deficiency predicted future disease course even amongst patients carrying the ATG16L1 T300A risk variant gene [74].

Brownson et al. [71] assessed 17 micronutrients in 216 adults with IBD receiving biologic therapy and prospectively followed patients for 12 months. Zinc deficiency was seen in 12.9% of patients. Plasma zinc concentrations correlated with CRP, faecal calprotectin and serum albumin. In CD, lower plasma zinc was associated with subsequent surgery and corticosteroid requirement and predicted the need for surgery on logistic regression [OR 0.67, 95% CI 0.47–0.96, *p* = 0.029]. No comparable association was observed in UC, and the associations were lost after adjustment using Cox proportional hazards modelling, suggesting much of the effect could be driven by systemic inflammation.

Not all studies have found an association between low plasma zinc and adverse IBD outcomes; Andres et al. [13] retrospectively assessed micronutrient deficiencies in 204 patients with IBD, comparing adults aged 18–59 years with adults ≥60 years. Zinc deficiency was seen in 37.4% of the overall cohort and was not significantly associated with corticosteroid use, intestinal complications or the composite adverse outcome, although non-significant trends were observed.

Overall, low circulating zinc is associated with adverse IBD outcomes in several observational cohorts, with the association appearing more consistent in CD than UC. However, the evidence remains heterogeneous, with studies varying in their definition of zinc deficiency, and several are limited by retrospective design, small sample sizes and low number of patients with zinc deficiency. Given the influence of systemic inflammation, hypoalbuminaemia and nutritional status on circulating zinc, reverse causality and confounding remain major limitations of the current evidence. Importantly, these associations do not establish a causal relationship between low zinc concentrations and adverse clinical outcomes.

### 4.4. Zinc Supplementation in IBD

The key clinical question is whether zinc supplementation and subsequent normalisation of serum or plasma zinc can improve IBD outcomes and therefore represent a potential therapeutic target. At present the clinical evidence is very limited. As summarised in Table 1, most existing studies are small and heterogeneous in design and often report surrogate markers of disease rather than clinically meaningful outcomes. Some publications also appear to derive from overlapping patient cohorts and should not be interpreted as independent replication. Collectively, these limitations make it difficult to establish whether zinc supplementation has a therapeutic benefit beyond correction of nutritional deficiency.

#### 4.4.1. Observational and Dietary Studies

Observational studies have demonstrated that zinc is a potentially modifiable factor. In the large retrospective cohort study reported by Siva et al. [11], subsequent normalisation of serum zinc within 12 months was associated with improved outcomes in both CD and UC. However, normalisation was assessed observationally rather than through a predefined supplementation protocol, meaning no conclusions regarding the role of zinc supplementation could be made, as it may merely have been a marker of improved inflammation.

Dietary studies have provided some indirect evidence but have not isolated a zinc-specific effect. Miyaguchi et al. [80] randomised 20 patients with mildly active UC to either an unrestricted diet or nutritional guidance promoting zinc intake alongside a Japanese diet rich in n-3 fatty acids. The patients receiving dietary advice demonstrated better clinical, endoscopic and histological outcomes. However, the small sample size and study design mean that the contribution of zinc relative to broader dietary changes could not be isolated. Similar dietary studies have demonstrated that higher dietary zinc is associated with a lower incidence of CD, but not UC [65]. Rizzello et al. [85] also reported a trend towards lower baseline dietary zinc intake in patients with active CD who did not respond to anti-TNFα therapy, although this did not reach statistical significance, and plasma zinc levels were not assessed.

#### 4.4.2. Zinc Supplementation

Human zinc supplementation studies in IBD are limited and difficult to compare due to substantial heterogeneity in study design, patient populations and supplementation regimens. There are considerable differences in zinc formulation and total elemental doses alongside differences in treatment duration and route of administration. Study populations also vary, including patients with active or quiescent disease, with some studies selecting patients with low circulating zinc concentrations and others not establishing baseline zinc deficiency. Furthermore, the endpoints assessed are inconsistent, and several studies rely on surrogate markers of disease activity rather than conventional clinical, biochemical or endoscopic outcomes. Several studies lack an appropriate comparator group, and some publications report overlapping patient cohorts. These differences limit comparisons between studies, meaning no meaningful conclusions regarding the effect size of zinc supplementation on IBD-related outcomes can be made.

In quiescent CD, zinc supplementation has been associated with improved markers of intestinal permeability [57] and normalisation of abnormal erythrocyte membrane long-chain fatty acid profiles [81]. However, studies in UC or mixed IBD cohorts have been inconsistent. Whilst zinc supplementation generally increases circulating zinc levels this has often not translated into improved markers of inflammation. In one mixed IBD group, zinc supplementation increased mucosal metallothionein concentrations but did not alter tissue superoxide dismutase activity, histological inflammation, albumin or disease activity [83]. In zinc-deficient patients with quiescent UC, supplementation with zinc gluconate corrected plasma zinc levels but did not increase erythrocyte superoxide dismutase activity [76]. Studies examining cytokine responses in UC have also been inconsistent. Zinc gluconate supplementation increased circulating zinc but did not significantly change TNF-α, IL-6 or IL-10, whilst a longer 60-day intervention reported reductions in IL-2 and IL-10 without consistent effects on IL-4, IL-6, IL-17, TNF-α or IFN-γ [77,78]. These findings suggest that zinc replacement can correct biochemical deficiency, but its effect on oxidative stress and systemic inflammation remains uncertain.

Clinical outcome data are even more limited. A placebo-controlled trial in 1977 demonstrated no clinical or endoscopic benefit from oral zinc sulphate supplementation in patients with active UC [75]. In contrast, rectal zinc-carnosine enemas showed significant improvement in endoscopic scores and induction of remission when used as an adjunct to standard induction therapy [79]. In a retrospective IBD cohort of zinc-deficient patients, oral zinc acetate improved serum zinc and was associated with reduced clinical disease activity in CD; however, no significant improvement in UC activity was observed [84]. The absence of a control arm limits the conclusions from this study.

Overall, the available interventional evidence remains insufficient to establish whether zinc supplementation improves clinical, biochemical or endoscopic measures of disease activity, or reduces the risk of treatment escalation, hospitalisation or surgery in patients with IBD.

## 5. Future Directions

Many unanswered questions remain regarding the role of zinc supplementation in IBD. It is unclear whether zinc supplementation should simply be viewed as replacement for confirmed deficiency as part of routine nutritional care, or whether it has a broader therapeutic role. Future studies should determine whether zinc supplementation in quiescent disease can reduce risk of relapses, or whether it offers additional benefit alongside conventional or advanced therapies during active inflammation. It is also unclear whether any potential benefit is limited to patients with a biochemical zinc deficiency, or whether it could extend to patients with an apparently normal circulating zinc concentration. A fundamental challenge is separating underlying zinc deficiency from the effects of active inflammation, hypoalbuminaemia and reduced dietary intake, alongside determining whether zinc itself has an independent role in intestinal inflammation.

Future studies should therefore examine zinc within a clearly defined clinical setting and move beyond biochemical correction alone. Placebo-controlled trials in patients with objectively low circulating zinc concentrations and otherwise quiescent IBD would reduce the confounding effect of inflammation and assess whether correction of low zinc status alters subsequent disease course. Outcomes should include clinically meaningful endpoints such as corticosteroid exposure, treatment escalation, IBD-related hospitalisation and surgery, supported by objective biochemical, endoscopic and/or histological measures of inflammatory activity. Zinc measurement and supplementation should also be standardised, including zinc formulation, elemental dose, treatment duration and adherence. Longer-duration studies should also monitor copper status and haematological indices. Detailed dietary analysis will also be important to quantify zinc intake accurately, alongside a meticulous nutritional assessment.

Although the association between low zinc and adverse outcomes appears stronger in CD than UC, few supplementation studies have focused specifically on CD, and most have used mechanistic rather than clinical endpoints. Malabsorption may partly explain this difference, particularly in patients with small bowel disease or previous intestinal resection, but this is unlikely to provide a complete explanation. Low zinc concentrations remain common in UC, and many patients in the available CD cohorts had isolated colonic, isolated ileal or ileocolonic disease rather than documented proximal small bowel involvement. Instead, low circulating zinc probably represents a composite marker of dietary intake, inflammatory burden, hypoalbuminaemia, diarrhoeal losses, previous surgery and overall disease severity. Future studies should determine whether any effect of supplementation differs according to baseline zinc status, disease phenotype and disease activity. Until further evidence is available, zinc should remain part of nutritional optimisation rather than be positioned as a therapeutic intervention.

## 6. Conclusions

Zinc deficiency and low circulating zinc concentrations are common in IBD and are associated with adverse clinical outcomes in several observational studies, particularly in CD. Experimental evidence provides biological plausibility through effects on epithelial barrier integrity, mucin production, Paneth-cell functions and immune signalling. However, circulating zinc is heavily influenced by inflammation and hypoalbuminaemia, and current evidence does not establish a causal relationship between zinc deficiency and adverse clinical outcome in IBD. Zinc deficiency should be identified and corrected as part of routine nutritional care; however, there is currently insufficient evidence that zinc supplementation improves IBD-related clinical outcomes. Well-designed studies are required to determine whether correcting zinc deficiency influences clinically meaningful outcomes in IBD.

## Figures and Tables

**Table 1:** Human interventional studies evaluating zinc interventions in IBD.

Author (Year)	Study Design	Disease Phenotype	N	Intervention ^†^	Comparator	Endpoint	Main Outcome	Limitations
Ulcerative colitis								
Dronfield (1977) [75]	R, DB, P	Active UC	51 (26 zinc, 25 placebo)	Oral zinc sulphate 220 mg three times a day ^‡^ for 4 weeks as adjunct to steroids and sulfasalazine	Active treatment (steroid and sulfasalazine) with placebo	Clinical and endoscopic improvement	No difference in clinical or endoscopic outcomes	Allowed co-treatment with steroids and sulfasalazine. Not zinc deficient at baseline
Matias (2015) [76]	OL	UC in remission. Zinc deficient.	24 (12 zinc deficient, 12 zinc replete)	Zinc deficient patients received zinc gluconate for 60 days (30 mg zinc/day as reported)	Zinc replete control group	Plasma & erythrocyte zinc and erythrocyte superoxide dismutase activity	Supplementation increased plasma zinc concentration with no change in superoxide dismutase activity	No placebo, open label, only patients in remission. No clinical endpoints
Soares (2018) [77] *	DB, NR	UC mixed disease	41 (23 zinc, 18 placebo)	Zinc gluconate 35 mg/day for 30 days (4.9 mg elemental zinc/day)	Placebo (in zinc replete patients)	Plasma and erythrocyte zinc, TNF-α, IL-6, IL-10; MT1G and ZIP-14 expression	Plasma zinc improved. No significant change in TNF-α, IL-6, IL-10. MT1G and ZIP14 expression decreased	Non-randomised, placebo group were zinc replete, small sample, mixed disease activity, no clinical endpoints
de Moura (2020) [78] *	DB, NR	UC mixed disease	41 (23 zinc, 18 placebo)	Zinc gluconate 35 mg/day for 60 days (4.9 mg elemental zinc/day)	Placebo (in zinc replete patients)	Plasma and erythrocyte zinc, serum cytokines, Mayo score at 30 and 60 days	Plasma/erythrocyte zinc improved, reduced Mayo score, IL-2 and IL-10 fell at 60 days, with no significant change in other cytokines	Non-randomised, placebo group were zinc replete, small sample, mixed disease activity, higher baseline Mayo in zinc deficient group, no repeat endoscopy
Itagaki (2014) [79]	R, P, SB	Mod–severe active UC	28 (18 zinc enema, 10 placebo)	Zinc-L-carnosine/polaprezinc enema, 150 mg (34 mg elemental zinc/day)	Placebo enema	Clinical response at week 1, endoscopic and histological improvement	Improved clinical response at week 1 (70.6% vs. 10%), improvement in endoscopic scores	Small sample, single centre, short follow up. Concurrent induction therapy. Formulation not comparable to oral zinc
Miyaguchi (2022) [80]	R	Mild active UC	20 (10 zinc rich diet, 10 unrestricted diet)	Nutritional guidance promoting zinc intake plus Japanese diet rich in n-3 fatty acids for 12–24 weeks	Unrestricted diet	Clinical remission at week 24. Clinical, endoscopic and histological outcome	Significantly lower CAI and UCEIS scores. Improved plasma zinc concentration	Broad dietary intervention that cannot isolate effect of zinc, small pilot study, no zinc supplementation, no placebo
Crohn’s disease								
Belluzzi (1994) [81]	OL	Quiescent CD	40 (20 CD, 20 controls)	Zinc sulphate 200 mg/day for 6 weeks (48.5 mg elemental zinc/day)	Non-supplemented healthy comparator	Plasma zinc, red cell phospholipid long-chain fatty acid and plasma fatty acid profile	Plasma zinc increased, and red cell fatty acid profiles improved; no change in plasma fatty acid profile	Uncontrolled study, no treatment or IBD comparator arm, no clinical endpoints
Sturniolo (2001) [57]	OL	Quiescent CD and abnormal intestinal permeability	12	Zinc sulphate 110 mg three times a day for 8 weeks (75 mg elemental zinc/day)	None	Plasma and urinary zinc concentrations. CDAI, clinical relapse, lactulose/mannitol permeability ratio	Plasma and urinary zinc increased. No significant effect on CDAI score. Lactulose/mannitol ratio improved	Small study, open label, no comparator arm
Mixed IBD								
Van de Wal (1993) [82]	R, DB, CO	Mixed IBD mild to moderate activity	13	Zinc aspartate 100 mg three times a day for 4 weeks (60 mg elemental zinc/day)	Placebo (cross over design)	Natural killer cell activity	Supplementation reduced peripheral blood natural killer cell activity	Small, mixed IBD cohort; no clinical, endoscopic or histological endpoint
Mulder (1994) [83] ^#^	DB, P, CO	Quiescent or moderately active IBD	14	Zinc aspartate 300 mg/day for 4 weeks (60 mg elemental zinc/day)	Placebo (cross over design)	Plasma zinc, MT and SOD concentrations, histological, albumin and disease activity	Plasma zinc increased, with slight mucosal MT elevation, but no change in SOD, histology, albumin or disease activity	Small study, short treatment duration, mild disease activity, minimal clinical outcome
Sakurai (2021) [84]	O, Ret	Zinc deficient UC or CD receiving zinc acetate hydrate	51 (CD = 40, UC = 11)	Zinc acetate hydrate 25–150 mg elemental zinc/day; assessed at 4 and/or 20 weeks	None	Serum zinc, partial Mayo (UC) or CDAI (CD)	Serum zinc improved, CDAI improved in CD, partial Mayo did not improve in UC	Retrospective, observational, uncontrolled, small study, no endoscopy, concurrent IBD care

CAI, clinical activity index; CD, Crohn’s disease; CDAI, Crohn’s Disease Activity Index; CO, cross-over; DB, double-blind; IBD, inflammatory bowel disease; L/M, lactulose/mannitol; MT, metallothionein; NK, natural killer; NR, non-randomised; O, observational; OL, open-label; P, placebo-controlled; R, randomised; Ret, retrospective; SB, single-blind; SOD, superoxide dismutase; UC, ulcerative colitis; UCEIS, Ulcerative Colitis Endoscopic Index of Severity. * Appears to report overlapping analyses from the same UC cohort and should not be interpreted as independent studies. ^†^ Elemental zinc equivalents are provided where reported in the original publication or reliably calculated from the specified formulation. Otherwise doses are presented as reported. ^‡^ elemental zinc content not specified in the original publication. ^#^ conducted by the same research group using similar zinc aspartate cross-over designs, with possible cohort overlap.

## Data Availability

No new data were created or analysed in this study. Data sharing is not applicable to this article.

## Abbreviations

The following abbreviations are used in this manuscript:

AKT	protein kinase B
CD	Crohn’s disease
CDAI	Crohn’s Disease Activity Index
CI	confidence interval
CRP	C-reactive protein
DSS	dextran sodium sulphate
IBD	inflammatory bowel disease
IFN	interferon
IL	interleukin
L/M	lactulose/mannitol ratio
MT	metallothionein
mTOR	mammalian target of rapamycin
NK	natural killer
OR	odds ratio
PI3K	phosphoinositide 3-kinase
SOD	superoxide dismutase
TNBS	trinitrobenzene sulphonic acid
TNF	tumour necrosis factor
UC	ulcerative colitis
UCEIS	Ulcerative Colitis Endoscopic Index of Severity
ZIP	Zrt-/Irt-like protein
ZnT	zinc transporter
ZO-1	zonula occludens-1
